# Editorial: Women in health services: Health policy and management 2021

**DOI:** 10.3389/frhs.2022.1014393

**Published:** 2022-09-09

**Authors:** Jacqueline Ponzo, Marjan van den Akker

**Affiliations:** ^1^Departamento de Medicina Familiar y Comunitaria, Facultad de Medicina, Universidad de la República, Montevideo, Uruguay; ^2^Escuela de Graduados, Facultad de Medicina, Universidad de la República., Montevideo, Uruguay; ^3^Institute of General Practice, Faculty of Medicine, Goethe University Frankfurt, Frankfurt, Germany; ^4^Academic Center for General Practice, Department of Public Health and Primary Care, KU Leuven, Leuven, Belgium; ^5^Department of Family Medicine, Maastricht University, Maastricht, Netherlands

**Keywords:** women, health management & policy, health service, research—development-innovation, research

Faced with some of the articles in this edition of Frontiers on the topic “*Women in Health Services: Health Policy and Management 2021*,” readers may wonder, where are the women? given that the research problem is not limited only to people of this gender in several of the included studies.

If you look closely, you will find that women are on both sides of the investigation. They appear explicitly as a research subject in only one of the papers (*Health-Related Challenges and Coping Strategies Among Women During Pandemics*), but they are as researchers in all of them (Sahay et al.). This introduces us to a new—necessary—perspective of critical reading: the analysis of authors with a gender perspective. Although a large and increasing part of students at medical faculties are female, their roles as first, second and last authors are still lagging behind. A similar gender gap is visible in health research funding, resulting in a disproportionate part of young female professionals leaving the academic field. The collection of papers in this special issue facilitates women in their role of leading academics and as such support them as role models.

A review of this selection shows that the femininity index in the set of 20 authors is 1.86. It is also interesting to note that in all cases the first author is a woman. Two of them are undergraduate medical students, two are doctoral students, and one is a researcher at a research center run by another woman.

Is this important? Can research have a gender? What is important in solving problems is the incorporation of the diversity of views. Not only because it is a matter of rights—and therefore it is necessary to ensure access to research and publication opportunities—, but also because the place of the gaze changes what is seen. The hegemonic science that has dominated the field of health has not considered this aspect for many decades, as if reality were a neutral and “celibate” matter. But in the field of health—as in any other, although perhaps more than others—, nothing is neutral. It is not about assigning sex or gender to the investigation, but about integrating perspectives. The gender perspective is necessary, it enriches. This small collection of articles is proof of that.

The issue that crosses these articles is the policy and management of health services. Within this, the dominant characteristic of the group is heterogeneity: the objects/subjects of study are diverse (a new quality indicator of the health system based on the general practitioners time available per patient per year, the relationship between burnout, workload in health professionals and the psychosocial safety climate, tools to improve communication with people with intellectual and developmental disabilities for equity in access to health, the lessons learned at the first level of care in the care of COVID-19 or coping strategies for women during this pandemic. The countries and areas where research was developed are also varied (the studies come from India, Switzerland, Benin, United Arab Emirates/United Kingdom and United States), although the first level of care and the community setting are repeated in almost all of them.

Into this diversity, there is also homogeneity. Research problems are, in all cases, peculiar in the sense that they are “small,” silent, “interstitial.” Perhaps that is where the gender perspective is most noticeable, women and health services, women looking at and investigating health services, the deep look, capable of investigating “small” things, those typical of social reproduction, those that are usually devoid of value in the market, but so necessary for life, as well as for health.

Another characteristic—by omission—worth noting: the absence of Latin American studies. This regional quota is provide in this topic by one of the editors—also the author of this editorial, but it is noteworthy that this advance in the visibility of the academic production of women, of their research on “interstitial” issues, of their work in community scenarios, emerging, often marginal to health systems, which occurs in many parts of the planet—and this topic shows it—, still does not cover Latin America in the same way. The language surely influences, although it is probably not the only thing. The proposal remains as a concern, to invite reflection, which allows us to continue advancing.

Meanwhile, it is worth pausing to observe some of the contributions made by these works. In *General Practitioner Time Availability Per Inhabitant Per Year: A New Indicator to Measure Access to Primary Care* (led by student Beer et al.), the proposed indicator is as simple as it is powerful. It synthesizes with simple and easily accessible data, aspects of structure and process of the health system. How much time can a GP devote to each person each year? A simple twist in the use of data generates a measure that allows the complexity of the system to be summarized and the center to be relocated to an issue that matters: the encounter, the listening. Barbara Starfield provided extensive evidence to document the benefits of a health system based on primary health care (1). Her “Primary Care Assessment Tool” (PCAT) remains current and is being updated to better assess health services (2). The proposed new indicator may be complementary to the powerful tool developed by Starfield.

“*Lessons Learnt From the Experiences of Primary Care Physicians Facing COVID-19 in Benin*” studied the working climate in primary health care in Benin, during the first year of the COVID-19 pandemic and reported high levels of stress and anxiety, as well as feelings of lacking training and coordination (Bello et al.). The insufficiencies found were not necessarily new ones, but became more prominent during the COVID-19 crisis.

The systematic review (qualitative studies) “*Health-Related Challenges and Coping Strategies Among Women During Pandemics*” takes a broader look: how the COVID-19 pandemic (and other pandemics) influence health-related challenges in female patients and female health care workers (Sahay et al.). Also here, we see the sharp increase in stress and anxiety, avoidance of reproductive care. At the same time women show high levels of resilience and adaptive capacities. For the continuity and quality of care, this is hopeful, since 70% of healthcare workers are female.

“*Psychosocial Safety Climate Moderates the Effect of Demands of Hospital Accreditation on Healthcare Professionals*” is an interesting study that explores job demands and resources and burnout/work engagement during hospital accreditation (Alshamsi et al.). This management process was found associated with the effects in health workers. More research is needed in this field.

The information transfer between patients, their caregivers, and their healthcare providers is associated with the disparities in health. The persons with intellectual and developmental disabilities (IDD) live 20 fewer years than the average person. “*Roadmap for Creating Effective Communication Tools to Improve Health Equity for Persons With Intellectual and Developmental Disabilities*” studies this problem and some tools developed for improving it (Dharampuriya and Abend).

We hope these publications will inspire you, for further research, policy and care with and for women, men and gender differences all over the world.

## Author contributions

JP developed the first draft. MvdA reviewed it, approved, and added her view in several paragraphs. Both commented the articles of the topic and added reflections about the role of the women in the health services and in research. Both reviewed the final version and agreed.

## Conflict of interest

The authors declare that the research was conducted in the absence of any commercial or financial relationships that could be construed as a potential conflict of interest.

## Publisher's note

All claims expressed in this article are solely those of the authors and do not necessarily represent those of their affiliated organizations, or those of the publisher, the editors and the reviewers. Any product that may be evaluated in this article, or claim that may be made by its manufacturer, is not guaranteed or endorsed by the publisher.
